# *Torque Teno Virus* in the vagina: potential influence on health and disease

**DOI:** 10.1016/j.clinsp.2026.101087

**Published:** 2026-09-05

**Authors:** Lillian Morgado Leitão, Steven S. Witkin, Tania Regina Tozetto-Mendoza, José Maria Soares-Jr, Iara Moreno Linhares

**Affiliations:** a Department of Obstetrics and Gynecology, Faculdade de Medicina da Universidade de São Paulo, São Paulo, SP, Brazil; b Department of Obstetrics and Gynecology, Faculdade de Medicina da Universidade de São Paulo, Laboratório de Ginecologia Estrutural e Molecular ‒ LIM-58, São Paulo, SP, Brazil; c Instituto de Medicina Tropical de São Paulo, Faculdade de Medicina da Universidade de São Paulo, Laboratório de Molecular Genetics ‒ LIM-46, São Paulo, SP, Brazil; d Department of Obstetrics and Gynecology, Weill Cornell Medicine, NY, USA; e Instituto de Medicina Tropical de São Paulo, Faculdade de Medicina da Universidade de São Paulo, Laboratório de Virologia ‒ LIM-52, São Paulo, SP, Brazil

**Keywords:** Torque teno virus, Vaginal immunity, Vaginal infections, Vaginal secretions

## Abstract

T*orque Teno Virus* (TTV) is one of the most prevalent dna viruses identified in the vagina. while its presence under a variety of conditions is undisputed, whether this is of functional relevance remains unresolved. in this review we question the prevailing assumption that TTV is an endogenous commensal virus with no role in health or disease at that site. we present data from published studies, and extrapolate from relevant related investigations, to suggest that TTV is likely an active contributor to both vaginal well-being and pathological processes. the host immune response to TTV is reviewed and how various aspects of this interaction may impact immunity to pathogenic microorganisms. whether the presence of TTV inhibits or potentiates the pathogenic potential of other viruses, as well as the possible involved mechanisms, is presented. properties of vaginal epithelial cells are described and the potential influences of TTV on their activities are delineated. Known differences in the composition of the vaginal bacterial microbiota are described and how these changes are reflected by concomitant alterations in the TTV level. Lastly, how detection of TTV in the vagina of female partners of infertile couples may be relevant to indicators of potential causation is reviewed. The sum of our efforts will hopefully encourage further TTV-related investigations, especially in women, and provide an enhanced understanding of the role of this virus in clinically relevant processes.

## Highlights

• Vaginal *Torque Teno Virus* levels influenced by microbiome and immune activation.
• *Torque Teno Virus* in vagina may help or hinder local immune protection.
• Classifying *Torque Teno Virus* as merely commensal in vagina remains premature.

## Introduction

The realization that multiple viruses reside in the vagina even in apparently healthy women is a recent occurrence. DNA and RNA viruses capable of infecting human cells, as well as viruses that infect bacteria, fungi and protozoal species that are present in the vagina, have been detected.^1^, ^2^, ^3^ The interaction of these viruses with human and microbial constituents of the vaginal lumen, and the consequences of these interactions for health and disease, remain largely a matter of speculation.

One of the most prevalent viral inhabitants of the human vagina is *Torque Teno Virus* (TTV). This virus has been reported in the vagina of approximately 50% of pregnant and non-pregnant women.^1^^,^^4^, ^5^, ^6^ TTV is the most frequently detected *Alphatorquevirus* and is a member of the *Anelloviridae* family. It is a small, single stranded circular DNA virus and, based on its ubiquitous presence in multiple body fluids and lack of association with pathogenic conditions, is considered a nonpathogenic endogenous virus.^1^^,^^2^

In this review we summarize studies that have detailed variations in the detection of TTV as well as its titer in the vagina in association with alterations in the vaginal environment. Based on these investigations we suggest that TTV exerts a multitude of effects on genital tract well-being and propose potential mechanisms by which TTV may either enhance or diminish local defense mechanisms. A generalized review on the potential of anelloviruses to contribute to a wide range of human diseases has recently been published.^7^

### TTV variations with vaginal microbiome composition

Studies relating the detection and titer of vaginal TTV to variations in the dominant bacterial species in the vagina have been mainly reported in pregnant women.^4^, ^5^, ^6^ In the majority of reproductive age women the vaginal bacterial microbiota is dominated by one of four species of *Lactobacilli: L. crispatus, L. iners, L. jensenii* or *L. gasseri.* In a minority of women levels of *Lactobacilli* in the vagina are drastically reduced or absent and a variety of other facultative or anaerobic bacteria predominate. Vaginal dominance by bacteria other than *L. crispatus, L. jensenii* or *L. gasseri* is associated with an elevated level of local inflammation, pro-inflammatory cytokine production and enhanced susceptibility to infection or pregnancy complications. The production by *L. crispatus, L. jensenii* and *L. gasseri* of both the d- and l- chiral isomers of lactic acid lowers vaginal pH to a level inhibitory to the maintenance and proliferation of many invasive pathogens. d-lactic acid has additional properties that aid in anti-microbial local immune defense.^8^ In contrast, *L. iners* is only capable of l-lactic acid production while the great majority of other vaginal bacteria do not produce either isomer. *L. crispatus* was the predominant *Lactobacillus* species in the vagina in the majority of women evaluated in the referenced studies. Concomitantly, local levels of pro-inflammatory molecules were lowest when *L. crispatus* was dominant. The evidence is persuasive that an *L. crispatus*-dominated vaginal microbiota corresponds to decreased immune activation and a general immune quiescence at this site. Regarding the association of vaginal bacterial microbiome composition with levels of TTV, the presence and concentration of TTV was shown to be significantly lower when *L. crispatus* prevailed compared to when *L. iners* or non-*Lactobacilli* predominated.^4^, ^5^, ^6^ Thus, the ability of TTV to persist and to effectively replicate in the vagina is influenced by the composition of the vaginal microbiome and the local level of immune activation. In low resource areas where determination of the vaginal bacterial composition and the level of local immune activity is not available, assessment of the TTV concentration in the vagina during pregnancy can serve as a substitute predictive biomarker for bacterial dominance and inflammation status.

### TTV-immune system interaction

The repertoire of TTV interactions with the host immune system has been recently reviewed and summarized.^9^ Regions in the TTV DNA contain what is known as CpG islands. These are areas of unmethylated DNA that are not typically present in the human genome. In the DNA of humans CpG is highly methylated. CpG islands are an example of a Pathogen-Associated Molecular Pattern (PAMP) that can be recognized by regions on mammalian cells called Pathogen Recognition Receptors (PRRs). The properties of CpG islands and their immune functions under different conditions has been the subject of an extensive review.^10^ The interaction of PAMPs with PRRs is a basic component of innate immunity and results in generation of immune system activation against the PAMP present in a multitude of pathogens. Toll-like Receptor (TLR)-9, is the PRR that specifically recognizes CpG islands in microbial DNA. It has been identified on vaginal epithelial cells^11^ in addition to being present on dendritic cells and macrophages. Thus, the persistent presence of TTV in the vagina can result in persistent activation of innate immunity and an enhanced protection against potential pathogens that enter that site. Interestingly, it has been reported that some CpG islands may, conversely, induce an inhibition of cell-mediated immunity. An area for future investigation would be to evaluate if different TTV isolates have opposing influences on TLR-9-directed immunity, and if this is determined by the composition of their CpG islands or neighboring nucleotides. Do divergent strains of TTV differ in their CpG frequency and/or composition and thus vary in their ability to compete for PPR binding with CpG islands present on the DNA of microbial pathogens and thereby inhibit pathogen recognition. The presence and/or titer and specific strains of TTV in the vagina may either aid or thwart the local protective immune response to microbial pathogens. This provides a reasonable explanation for variations in TTV-related associations across different populations.

Whether TTV induces a humoral immune response that aids in its removal or inactivation is controversial. Antibodies in the systemic circulation that are reactive with TTV surface antigens have been reported by some investigators but not confirmed by other reports.^9^^,^^12^ It remains undetermined whether IgG antibodies to TTV derived from the blood are transduced into the vagina. Similarly, whether TTV induces the local production of IgA antibodies in the genital tract can be readily determined but to date remains unknown. It is reasonable to speculate that variations in anti-TTV IgA or IgG antibody levels in the vagina of individual women may differentially affect the duration and/or direction of TTV-related activity.

### TTV and vaginal epithelial cells

TTV has been shown to be present in ciliated respiratory tract epithelial cells.^13^ The concomitant ability of TTV to infect and persist within epithelial cells in the vagina remains untested. Similarly, it remains to be determined whether the presence of TTV can positively and/or negatively influence any of the myriad functions of vaginal epithelial cells. Evidence had been presented that the vaginal epithelium is a major contributor to immune defense mechanisms at this site.^14^ These cells release immune-reactive components that promote local anti-microbial activity. Epithelial cell-derived immune activity is lowest when *L. crispatus* predominates in the vaginal microbiota and becomes markedly elevated when *L. iners* or non-*Lactobacillus* species are in the majority. Vaginal epithelial cells also bind and release physiologically active compounds that influence the local external environment. Their intake and subsequent release of glycogen into the vaginal lumen determines to a large extent the competitive ability of different bacteria to persist and proliferate. Norepinephrine is released into the circulation in times of stress. Its subsequent binding to vaginal epithelial cells up-regulates the local immune response to external stress. Autophagy is induced in vaginal epithelial cells when their membranes are penetrated by bacteria or viruses. This basic intracellular process results in the engulfment and destruction of these microorganisms. The extent of vaginal epithelial cell autophagy is variable, depending on the relative concentration of different microorganisms that co-exist in the external environment.^15^^,^^16^ The influence of TTV on clinically relevant functions of vaginal epithelial cells remains untested and deserves to be addressed.

### TTV and exposure to infection

Another pertinent area of investigation is whether the consequences of TTV’s presence differ between healthy women and in those whose immunity is altered due to the presence of an infection. It has been shown in mice that the presence of an innocuous commensal virus alters and increases the negative consequences of a subsequent infection.^17^ Evidence has been presented that this “double hit hypothesis” may also be relevant to TTV infection. TTV levels in the cervix have been shown to be increased in the presence of a Human Papillomavirus (HPV) infection^18^ and TTV is detected more frequently in women positive for high risk HPV types than in those with low risk HPV types.^19^ Similarly, TTV levels are elevated in respiratory tract samples from individuals with a variety of respiratory tract infections.^20^ It has also been suggested that TTV functions as a co-factor in the initiation and development of other viral infections.^21^ Does the presence of TTV lower the threshold for an immune response to pathogens? Is a subclinical infection more likely to induce a detectable and deleterious immune response if TTV is also present? Alterations in host defense or local microbial composition may lead to TTV becoming an opportunistic abettor of pathogenic changes. Thus, the TTV level may be a sensitive biomarker of viral infections as well as exerting a potential adjuvant effect on pathogen efficacy. This requires further investigation.

### TTV and pregnancy

Does the vaginal TTV titer indicate the occurrence of pregnancy complications? A recent exploratory metagenomic investigation evaluated the presence of vaginal TTV in women undergoing a cycle of *In Vitro* Fertilization (IVF).^22^ In 34 women undergoing IVF for infertility 47% were positive for TTV. Interestingly, TTV was most prevalent in women who could not conceive due to an ovulation disorder or in those whose male partners were defective in sperm production. In men with deficient sperm parameters an increased level of TTV in their semen may be related to excess immune system activation in the male genital tract that is then transmitted to the female partner during sexual intercourse. Alternatively, atypical semen may be at increased likelihood of inducing inflammation and thus elevated TTV production in the female recipient. A single study of 10 drug-addicted males with TTV in their systemic circulation detected TTV in the semen of 60% of the men.^23^ This provides direct evidence that TTV can be sexually transmitted from males to females. Indirect evidence of this occurrence comes from a study that reported an association between TTV prevalence in women and their number of lifetime sexual partners.^24^ The association of TTV with a failure of ovulation is consistent with altered genital tract immunity as contributory to an ovarian disturbance. Studies to relate the TTV level in semen with various sperm parameters appears warranted.

A study of pregnant women undergoing *in utero* fetal surgery to correct a neurological defect in their fetus tested by metagenomics for TTV in amniotic fluid that was obtained prior to surgical intervention. The detection of TTV correlated with an earlier gestational age at subsequent delivery and respiratory distress in the newborn, as compared to women negative for amniotic fluid TTV.^25^ A Japanese study of ten TTV-serum positive pregnant women with healthy outcomes reported that all were negative for TTV in their amniotic fluid.^26^ Thus, although reliable conclusions cannot be derived from two preliminary reports, the detection of TTV in amniotic fluid might indicate adverse intraamniotic conditions and be of clinical relevance.

TTV in the maternal circulation is more common in women with prior pregnancies than in nulliparous women.^27^^,^^28^ Pregnancy-induced immune system alterations that persist post-partum apparently influence the replicative ability of TTV. This may be due to the proliferation of regulatory T-cells that initially appear in the maternal circulation during pregnancy. The level of these cells increases with each subsequent gestation and persists following parturition. Alteration in the magnitude of immune responses by these regulatory T-cells may promote TTV persistence. Alternatively, the increased elevated level of endogenous inflammation present in parous women can also result in a greater level of TTV proliferation.

### Clinical implications

TTV is present in the vagina of many women in apparent good health. This precludes the value of testing for this virus in the vagina in all women. However, detection of elevations in the vaginal TTV titer has clinical implications. It functions as a biomarker signal for the presence of an elevated localized pro-inflammatory immune response and highlight the need for subsequent evaluation for infections or autoimmune anomalies in both pregnant and non-pregnant women. In addition, in an infertility evaluation elevations in TTV could point to the need to examine semen parameters in the male partner. Furthermore, in pregnancies suspected of complications related to infection or sterile inflammation analysis of amniotic fluid for TTV may be beneficial in aiding this diagnosis.

## Conclusion

The presence of TTV in the vagina may be innocuous, beneficial or deleterious, depending on factors such as the specific TTV strain that is present, the composition of the vaginal bacterial microbiome, simultaneous or subsequent exposure to pathogenic microorganisms or host-related variables. We assert that it remains premature to classify TTV as merely a commensal nonpathogenic inhabitant of the vagina. Additional experimentation may help in defining the local conditions that determine the consequences of TTV’s presence. This may lead to development of novel protocols to enhance the vaginal environment and improve women’s health.

## Statement of ethics

This review of previously published articles does not require ethical approval.

## Authors’ contributions

Conceptualization: Steven S. Witkin, Iara Moreno Linhares.

Methodology: Lillian Morgado Leitão, Steven S. Witkin, Tania Regina Tozetto-Mendoza, José Maria Soares-Jr, Iara Moreno Linhares.

Validation: Lillian Morgado Leitão, Steven S. Witkin, Tania Regina Tozetto-Mendoza, José Maria Soares-Jr, Iara Moreno Linhares.

Writing-original draft: Lillian Morgado Leitão, Steven S. Witkin, Iara Moreno Linhares.

Review and editing: Lillian Morgado Leitão, Steven S. Witkin, Tania Regina Tozetto-Mendoza, José Maria Soares-Jr, Iara Moreno Linhares.

## Funding

The authors did not receive any funding during this study.

## Declaration of competing interest

The authors declare no conflicts of interest.

## Data Availability

The datasets generated and/or analyzed during the current study are available from the corresponding author upon reasonable request.
